# Gut microbiota, determined by dietary nutrients, drive modification of the plasma lipid profile and insulin resistance

**DOI:** 10.1016/j.isci.2021.102445

**Published:** 2021-04-16

**Authors:** Yoshiyuki Watanabe, Shiho Fujisaka, Kazutaka Ikeda, Masaki Ishikawa, Takahiro Yamada, Allah Nawaz, Tomonobu Kado, Takahide Kuwano, Ayumi Nishimura, Muhammad Bilal, Jianhui Liu, Kunimasa Yagi, Koji Hase, Kazuyuki Tobe

**Affiliations:** 1 First Department of Internal Medicine, Faculty of Medicine, Academic Assembly, University of Toyama, 2630 Sugitani, Toyama 930-0194, Japan; 2 Laboratory of Biomolecule Analysis, Department of Applied Genomics, Kazusa DNA Research Institute, 2-6-7 Kazusa-kamatari, Kisarazu, Chiba 292-0818, Japan; 3 Laboratory of Clinical Omics Research, Department of Applied Genomics, Kazusa DNA Research Institute, 2-5-23 Kazusa-kamatari, Kisarazu, Chiba 292-0818, Japan; 4 Division of Biochemistry, Faculty of Pharmacy and Graduate School of Pharmaceutical Sciences, Keio University, Minato-ku, Tokyo 105-8512, Japan; 5 Department of Molecular and Medical Pharmacology, Faculty of Medicine, Academic Assembly, University of Toyama, 2630 Sugitani, Toyama 930-0194, Japan

**Keywords:** Microbiology, Microbiome, Lipidomics

## Abstract

The gut microbiota metabolizes the nutrients to produce various metabolites that play crucial roles in host metabolism. However, the links between the microbiota established by different nutrients and the microbiota-influenced changes in the plasma lipids remain unclear. Diets rich in cornstarch, fructose, branched chain amino acids, soybean oil (SO), or lard established a unique microbiota and had influence on glucose metabolism, which was partially reproduced by transferring the microbiota. Comparison of plasma lipidomic analysis between germ-free and colonized mice revealed significant impacts of the microbiota on various lipid classes, and of note, the microbiota established by the SO diet, which was associated with the greatest degree of glucose intolerance, caused the maximum alteration of the plasma lipid profile. Thus, the gut microbiota composed of dietary nutrients was associated with dynamic changes in the lipids potentially having differential effects on glucose metabolism.

## Highlights

• Diets with different nutrient compositions differentially affect glucose metabolism
• Gut microbiota established by soybean oil-rich (SO) diet impairs glucose metabolism
• Gut microbiota established by diets has dynamic effects on the plasma lipid profile
• SO diet has the greatest impact on the plasma lipid profile through gut microbiota

## Graphical abstract

**Figure:**
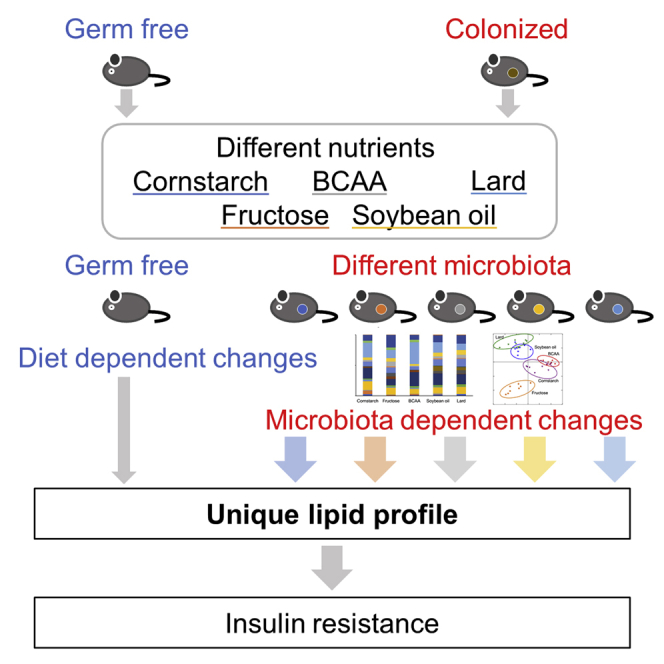

## Introduction

In recent years, it has become clear that the gut microbiota are one of the major regulatory factors of the host metabolism (Nicholson et al., 2012). Bacterial metagenomic analyses and gnotobiotic studies of obese diabetic subjects have demonstrated that dysbiosis can induce obesity and glucose intolerance (Turnbaugh et al., 2008) (Foley et al., 2018) (Cani et al., 2008). The composition of the microbial community in the gut is determined by multiple factors, including the diet, intake of antibiotics, comorbidities, and host genetic background (Ussar et al., 2015) (Fujisaka et al., 2016), and the species and their proportions are also unique to each individual. Therefore, it is difficult to identify any specific bacterial species as a determinant of the development of obesity or abnormal glucose metabolism. On the other hand, the mechanisms of actions of the metabolites produced by the entire microbiota could be common to individuals. The gut microbiota actively metabolizes nutrients as sources of energy, which results in the production of various metabolites (Nicholson et al., 2012). Short-chain fatty acids (FAs) , which are microbial metabolites of indigestible dietary fiber, have been reported to promote GLP-1 secretion and exert anti-inflammatory effects by activating the GPR 43 and GPR 41 receptors (Natarajan and Pluznick, 2014) (Brown et al., 2003) (Lin et al., 2012). The gut microbiota also dehydroxylate cholesterol-derived primary bile acids, converting them into secondary bile acids, which induce GLP-1 release to promote insulin secretion and increase the basal metabolic rate via TGR5 activation (Brighton et al., 2015) (Islam et al., 2011) (Watanabe et al., 2006) (Thomas et al., 2009) (Perino and Schoonjans, 2015). Thus, some bacterial metabolites are absorbed into the host circulation and act as signaling molecules to modulate energy and glucose metabolism. More evidence to suggest that the gut microbiota exert an important influence on metabolism is that germ-free (GF) mice show resistance to the development of obesity and glucose intolerance (Rabot et al., 2010). One of the underlying mechanisms is believed to be that in the absence of the gut microbiota, GF mice cannot utilize energy sources, such as short-chain FAs. However, comparisons between GF and conventional mice, reared in normal specific-pathogen-free (SPF) breeding environments, by mass spectrometry (MS)-based metabolomics have revealed that the gut microbiota regulate the levels of various host metabolites, and at least 145 metabolites were detected that were unique to conventional mice (Wikoff et al., 2009). In addition, untargeted metabolomic analyses have revealed that mice of different genetic and environmental backgrounds administered high-fat diets or antibiotics showed establishment of unique microbial compositions and diverse plasma metabolite profiles. Some of the metabolites were quantitatively correlated with the degree of insulin resistance and specific bacterial species, indicating that important cross talk occurs between the gut microbiota-derived metabolites and insulin resistance (Fujisaka et al., 2018).

Both genetic and environmental factors determine the host-specific composition of the gut microbiota (Korach-Rechtman et al., 2019);however, the most important driver is the dietary nutrient composition. In particular, lipids are metabolized by the gut microbiota, whereas the host also produces lipids. At present, it still remains unclear how regulation of the plasma lipid profile is directly modified by the gut microbiota. In addition, because the physiological environment, such as immunity and intestinal development of GF mice, are different from those of conventional mice (Tlaskalová-Hogenová et al., 2004), clarification of the underlying mechanisms is not possible by a simple comparison of GF and conventional mice. To clarify the exact influence of the gut microbiota on the plasma lipid profile, plasma lipidomic analysis was performed on GF mice and their littermates, so as to match for the genetic background and physiological conditions, colonized with the microbiota of chow-fed mice. Even though the lipid sources of the diets were similar, the plasma lipid profiles differed significantly among the mice fed the cornstarch (CS), Fru, and branched chain amino acid (BCAA) diets. Furthermore, the mouse group fed the soybean oil (SO) diet, which was the strongest inducer of insulin resistance, showed increases in the levels of a larger number of lipids when compared with the GF mice. Thus the gut microbiota established by dietary nutrients drive the plasma lipid composition, which interacts with different metabolic responses.

## Results

### Diets with different nutrient compositions differentially affected energy and glucose metabolism

**Figure 1:**
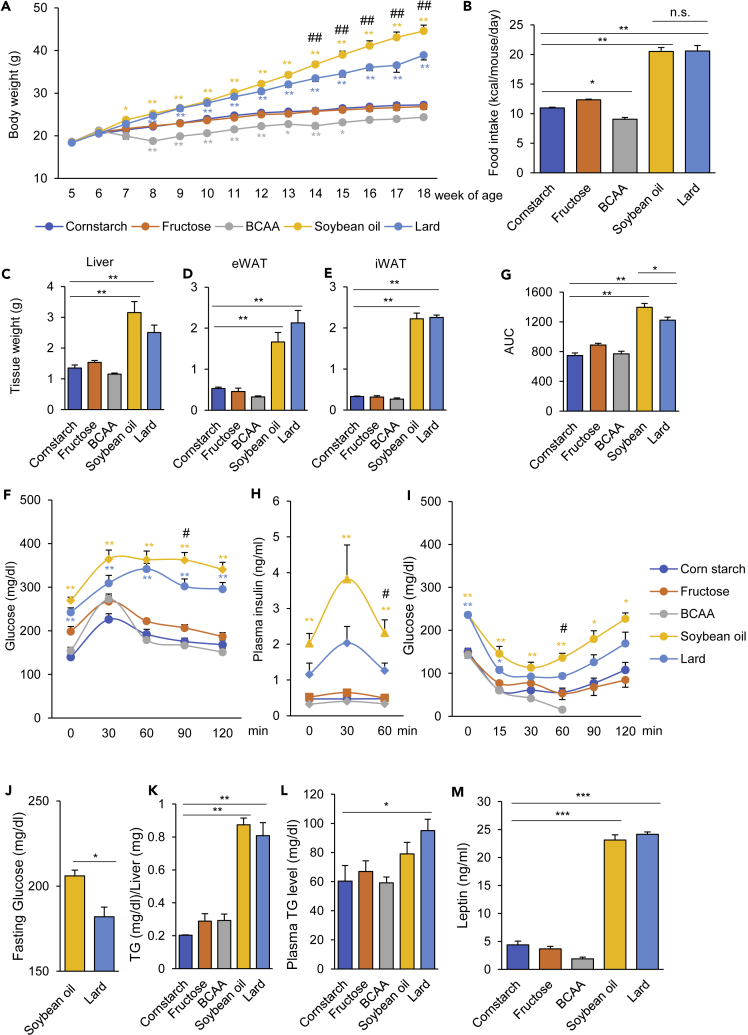
Diets containing different nutrient compositions have differential effects on the energy and glucose metabolism of mice (A and B) (A) Body weights and (B) food intakes of mice fed a diet rich in cornstarch (CS), fructose (Fru), branched chain amino acids (BCAA), soybean oil (SO), or lard (n = 8–9/group). ∗p < 0.05, ∗∗p < 0.01, comparison of mice fed the CS diet and each diet by ANOVA, followed by Tukey-Kramer's post hoc test. ##p < 0.01, comparison of mice fed the SO diet and the Lard diet by ANOVA, followed by Tukey-Kramer's post hoc test. (C–E) (C) Weights of the liver, (D) eWAT, and (E) iWAT in the mouse groups fed one of the experimental diets each for 21 week (n = 4–5/group). (F and G) (F) Blood glucose levels and (G) AUCs during the oral glucose tolerance test (OGTT) in the mouse groups fed one of the experimental diets each for 11−12 weeks (n = 8–9/group). ∗p < 0.05, ∗∗p < 0.01, comparison of mice fed the CS diet and each diet by ANOVA, followed by Tukey-Kramer's post hoc test. #p < 0.05, comparison of mice fed the SO diet and the Lard diet by ANOVA, followed by Tukey-Kramer's post hoc test. (H) Plasma insulin levels during the OGTT (n = 4–5/group). ∗p < 0.05, ∗∗p < 0.01, comparison of mice fed the CS diet and each diet by ANOVA, followed by Tukey-Kramer's post hoc test. #p < 0.05, comparison of mice fed the SO diet and the Lard diet by ANOVA, followed by Tukey-Kramer's post hoc test. (I) Results of the insulin tolerance test (ITT) in the mouse groups fed one of the experimental diets each for 13 weeks (n = 4–5). ∗p < 0.05, ∗∗p < 0.01, comparison of mice fed the CS diet and each diet by ANOVA, followed by Tukey-Kramer's post hoc test. #p < 0.05, comparison of mice fed the SO diet and the Lard diet by ANOVA, followed by Tukey-Kramer's post hoc test. (J) Fasting blood glucose levels in the mice fed the SO or Lard diet for 6 weeks. (K) Hepatic TG contents in the mouse groups fed one of the experimental diets each for 33 weeks (n = 4–5/group). (L) TG and (M) leptin levels in the plasma of the mouse groups fed one of the experimental diets each for 21 week (n = 4–5/group). ∗p < 0.05, ∗∗p < 0.01, ∗∗∗p < 0.001 by ANOVA, followed by the Tukey-Kramer or Dunnett post hoc test.

To investigate the effects of dietary interventions on the metabolic profiles in mice, we designed five different diets, namely, diets rich in cornstarch, fructose, BCAA, soybean oil, and lard (CS, Fru, BCAA, SO, and Lard diets, respectively) (Table 1). In the Fru diet, CS was replaced with fructose. The BCAA diet was supplemented with valine, leucine, and isoleucine to increase the protein-derived energy to 43%. The energy from fat and the FA compositions of the CS, Fru, and BCAA diet were almost the same. The two high-fat diets (SO and Lard) provided 60% of the energy from fat. The SO diet contained high amounts of ω6 polyunsaturated FAs, such as C18:2 and C18:3, whereas the Lard diet contained saturated FAs, such as C16:0 and C18:0 (Figure S1). As previously reported, the mice fed the SO and Lard diets (SO and Lard groups), both high-fat diets, gained more weight than those that were fed the CS diet (CS group), with increased food intake (Figures 1A and 1B). After 8 weeks (14 weeks of age), the weight gain in the SO group was significantly higher than that in the Lard group (Figure 1A). The mice fed the Fru diet (Fru group) showed no difference in either the amount of food intake or the body weight when compared with the CS group. The mice fed the BCAA diet (BCCA group) showed less weight gain, with decreased food intake (Figures 1A and 1B). The weights of the liver, epididymal white adipose tissue (eWAT), and inguinal white adipose tissue (iWAT) were significantly higher in both the high-fat diet (SO and Lard) groups, although they did not differ significantly between these two diet groups (Figures 1C–1E). The oral glucose tolerance test (OGTT) and insulin tolerance test showed impaired glucose tolerance with decreased insulin sensitivity in both the SO and Lard groups, although the glucose tolerance was significantly worse in the SO group, along with more elevated plasma insulin levels (Figures 1F–1I). The BCAA diet group was the most insulin sensitive (Figure 1I). The glucose intolerance induced by the SO diet was observed at 12 weeks of age, before the difference in body weight became apparent (Figure 1J). The hepatic triglyceride contents were significantly higher in both the high-fat diet (SO and Lard) groups, although there was no significant difference between these two groups (Figure 1K). Plasma TG levels were significantly elevated only in the Lard group (Figure 1L). The SO and Lard diets increased the plasma leptin levels, suggesting that they induced leptin resistance (Figure 1M). Ghrelin levels were higher in the Lard group compared with the CS group, but not significantly different from the SO group (Figure S2). These results suggest that both the high-fat diets (SO and Lard) induced obesity and insulin resistance, but the metabolic disorder was worse in the SO group than in the Lard group, independent of the body weight and the appetite. Thus, not only the caloric intake but also the lipid composition of the diet exerted a significant impact on the metabolic state.

**Table 1:** Nutrient compositions of each of the experimental diets

	Carbohydrate		Protein	Fat	
	Cornstarch	Fructose	BCAA	Soybean oil	Lard
Carbohydrate (kcal%)	**72**	**72**	47	18	18
Protein (kcal%)	18	18	**43**	22	20
Fat (kcal%)	11	11	11	**60**	**60**
Kcal/g	3.8	3.8	3.8	5.1	5.1
**Component (g)**					

Casein	200	200	200	200	200
Amino acids			**Valine** **Leucine** **Isoleucine**		
Soybean oil	25	25	25	**192.3**	25
Lard	20	20	20	75	**242.3**
Cornstarch + maltodextrin	**700**	0	450	200	200
Fructose	0	**700**	0	0	0
Cellulose	50	50	50	50	50

Bold numbers indicate high levels of the nutrient.

### High-fat diets induced chronic inflammation, but the chronic inflammation did not appear to be the sole determinant of insulin resistance in the SO group

**Figure 2:**
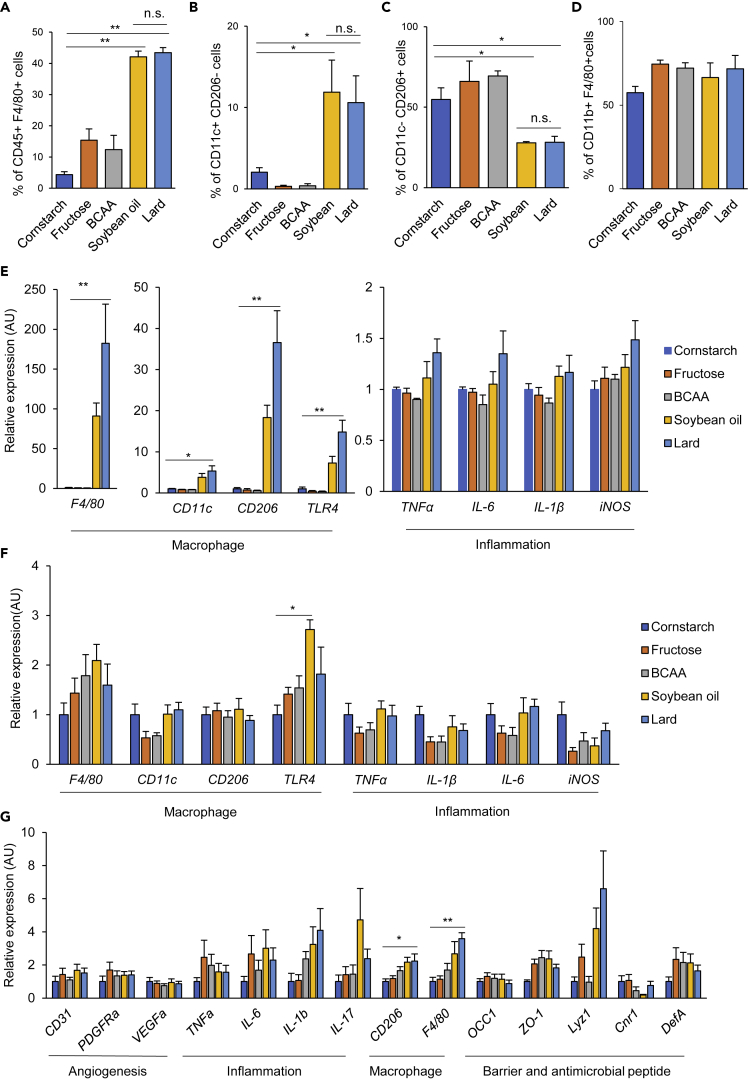
High-fat diet-induced inflammation in the adipose tissue and liver, although the inflammation appeared to account only in part for the higher insulin resistance observed in the SO group (A–C) Percentages of CD45+ F4/80+ macrophages (A), CD45+ F4/80+ CD11c+ CD206- M1 macrophages (B), and CD45+ F4/80+ CD11c- CD206+ M2 macrophages (C) in the eWAT of the mouse groups fed one of the experimental diets each for 21 weeks, as assessed by flow cytometry. (D) Percentages of F4/80+ CD11b+ macrophages in the livers of the mouse groups fed one of the experimental diets each for 21 weeks. (E and F) qPCR analysis of markers of macrophages and inflammation-related genes in the eWATs (E), livers (F), and colons (G) of the mouse groups fed one of the experimental diets each for 21 weeks (n = 4–5/group). ∗p < 0.05, ∗∗p < 0.01 by ANOVA, followed by Dunnett post hoc test. (G) qPCR analysis of angiogenesis-, inflammation-, macrophage-, and intestinal barrier-related genes in the colon of the mouse groups fed one of the experimental diets each. ∗p < 0.05, ∗∗p < 0.01 by ANOVA, followed by the Tukey-Kramer post hoc test.

Chronic inflammation is recognized as a major cause of insulin resistance in diet-induced obesity, and tissue macrophages comprise the major population of immune cells involved in this inflammation. Flow cytometric analysis of the adipose tissue revealed greatly increased proportions of F4/80+ macrophages and CD11c+ CD206- M1 macrophages and significantly decreased proportions of CD11c- CD206 + M2 macrophages in both the SO and Lard groups (Figures 2A–2C). Evidence of adipose tissue inflammation in the Lard group included increased expressions of macrophage and inflammation markers, with a similar trend also seen in the SO group (Figure 2E). In the liver, the proportions of CD11b+ F4/80+ macrophages were slightly increased in the Fru, BCAA, SO, and Lard groups, although the differences were not significant (Figure 2D). Only moderate effects of diet on the hepatic gene expressions were observed, except for increased expression of *TLR4* in the SO group (Figure 2F). In the colon, whereas the expressions of resident macrophage markers such as *CD206* and *F4/80* were increased in the Lard group, no alterations in the expressions of inflammation-related genes were evident. Expressions of genes encoding a tight junction-related marker, antimicrobial peptides, and angiogenesis-related genes were not altered in any of the diet groups (Figure 2G). In addition, we evaluated the intestinal permeability *in vivo* by the fluorescein isothiocyanate-dextran assay. Although there was a trend toward increased intestinal permeability in the SO and Lard groups, the difference did not reach statistical significance (Figure S3), suggesting that each diet had little effect on the gut barrier function in our study. Taken together, both the SO and Lard diets induced inflammation in the adipose tissue and the liver, thereby promoting insulin resistance. A greater degree of insulin resistance was observed in the SO group when compared with the Lard group. However, chronic inflammation did not appear to be the sole determinant of the higher degree of insulin resistance observed in the SO group, as there was no difference in the degree of inflammation between the two high-fat diet groups.

### Gut microbiota established by the SO diet impairs glucose metabolism

**Figure 3:**
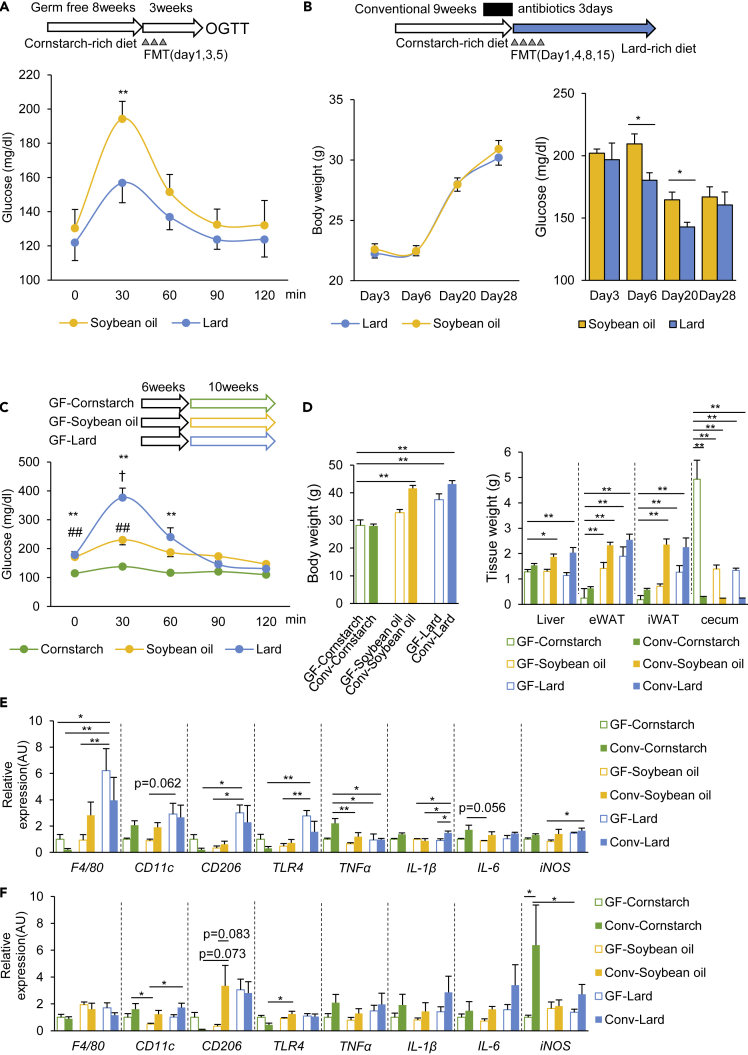
The gut microbiota established by the SO diet worsened the glucose metabolism in the host mice (A) Schematic overview of the study design for fecal microbiota transfer (FMT) to germ-free (GF) female mice, and the results of the OGTT performed 3 weeks after the FMT (n = 5–8). (B) Schematic overview of the study design for FMT to conventional SPF mice, and the body weights and random blood glucose levels of the recipient mice after the first FMT (n = 6–7). ∗p < 0.05, ∗∗p < 0.01 by an unpaired, two-tailed Student's t test. (C) Results of the OGTT in the GF mice fed the CS, SO, or Lard diet for 10 weeks (n = 4–5). ∗∗p < 0.01, comparison of GF mice fed the CS and Lard diets. ##p < 0.01, comparison of GF mice fed the CS diet and SO diets, †p < 0.05, comparison of GF mice fed the SO and Lard diets, followed by the Tukey-Kramer post hoc test. (D) Body weights and tissue weights of the GF and Col mice fed the CS, SO, or Lard diet for 10 weeks. (E and F) qPCR for macrophage and inflammation markers in the liver (E) and eWAT (F). ∗p < 0.05, ∗∗p < 0.01 by ANOVA, followed by the Tukey-Kramer post hoc test.

To determine whether the metabolic differences between the SO and Lard groups were related to the gut microbiota, we transferred the gut microbiota of the SO and Lard mice into GF mice. OGTT performed 3 weeks after the fecal microbiota transplantation (FMT) revealed that the recipient mice colonized with bacteria from the SO diet group showed significantly worse glucose tolerance than those that were colonized with bacteria from the Lard group (Figure 3A). To investigate the effects of FMT on the conventional SPF mice, the endogenous microbiota in the conventional mice were depleted by 3-day treatment with an antibiotic cocktail, and FMT was performed either from the SO group or the Lard group, followed by feeding of the mice with the Lard diet (Figure 3B). The mice that received the microbiota from the SO group showed increased random blood glucose levels when compared with those that received the microbiota from the Lard group, with no change of the body weight (Figure 3B). To further explore the contribution of the gut microbiota, GF mice were fed the CS, SO, or Lard diets (GF-CS, GF-SO, and GF-Lard groups, respectively) for 10 weeks. OGTT revealed that the GF-SO group showed slightly elevated blood glucose levels when compared with the GF-CS group, but lower levels when compared with the GF-Lard group. Thus the worse glucose tolerance induced by the SO diet than the Lard diet is greatly alleviated without microbiota, indicating an important contribution of the microbiota established by the SO diet to glucose metabolism (Figure 3C). Both the SO diet- and the Lard diet-fed GF mice gained less body weight and showed smaller weights of the liver, eWAT, and iWAT than the corresponding conventional mice. The cecum weights were larger in all the GF groups, suggesting that the microbiota actively metabolized and promoted absorption of the nutrients in the diet (Figure 3D). In the adipose tissue, macrophage and inflammatory gene expressions, such as *F4/80*, *TLR4,* and *IL-1β*, were increased in the GF-Lard group, but not in the GF-SO group (Figure 3E). The changes were relatively small in the liver (Figure 3F). Thus the gut microbiota established by the SO diet worsened glucose metabolism, while having little effect on tissue inflammation.

### Each diet established a unique bacterial community

**Figure 4:**
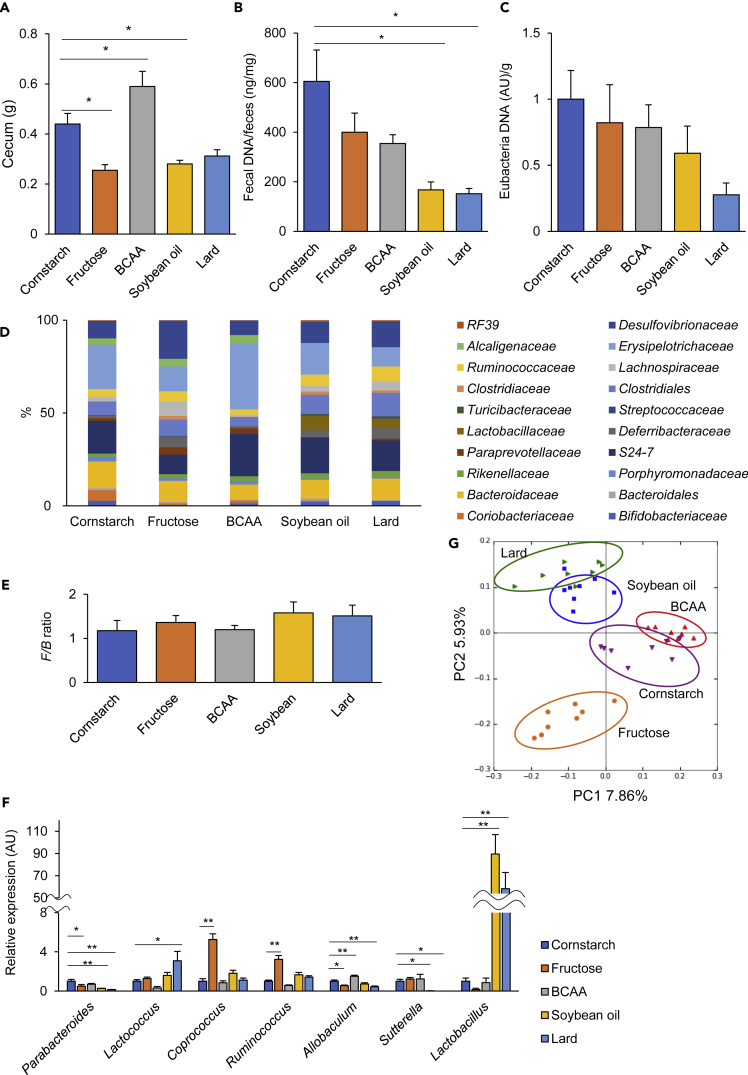
Dietary nutrient composition is the major determinant of the gut microbiota (A–C) (A) Cecum weights, (B) total DNA concentrations, and (C) eubacterial DNA levels in the fecal samples collected from the mouse groups fed one of the experimental diets each for 18 weeks (n = 8–9). (D and E) (D) Microbial compositions at the level of the family. (E) Average *Firmicutes/Bacteroidetes* ratio (*F/B* ratio) (n = 8–9). (F and G) (F) Examples of genera that showed significant differences, and (G) PCA of the fecal 16S rRNA sequencing data in 18-week-old mice. ∗p < 0.05, ∗∗p < 0.01 by ANOVA, followed by the Tukey-Kramer or Dunnett post hoc test.

The cecum sizes of the mice fed the Fru, SO, and Lard diets were smaller than those of the mice fed the CS diet, whereas those of the mice fed the BCAA diet were larger (Figure 4A). Both the total DNA levels and the relative eubacterial DNA levels in the feces were lower in the SO and Lard diet groups (Figures 4B and 4C), suggesting that each diet establishes a different bacterial biomass and fermentation activities. 16S rRNA sequencing analysis of the fecal samples was performed to determine the bacterial composition after 12 weeks on each diet. Overall, the gut microbiota was dominated by *Firmicutes* and *Bacteroidetes*. The Fru group showed increased counts of *Proteobacteria* and decreased numbers of *Actinobacteria*, whereas the BCAA group showed lower counts of *Deferribacteres*. The BCAA, SO, and Lard groups showed higher populations of *Firmicutes* (Figure S4). Looking at family level, mice fed the Fru diet increased Desulfovibrionaceae, Lachnospiraceae, and Deferribacteraceae, whereas those fed the BCAA diet showed increased Erysipelotrichaceae when compared with the other diet groups. Both the SO and Lard diets eliminated Alcaligenaceae and increased Lactobacillaceae. The proportions of Erysipelotrichaceae and Bacteroidales were lower in the Lard diet than the SO diet (Figure 4D). The *Firmicutes/Bacteroidetes* (F/B) ratio was elevated in both the high-fat diet groups, associated with a decrease in the numbers of Bacteroidetes (Figure 4E). At genus level, the SO and Lard diets decreased *Parabacteroides*, *Allobaculum* and *Sutterella*, and increased proportions of *Lactobacillus*. The Fru diet increased numbers of *Coprococcus* and *Ruminococcus*, and decreased numbers of *Parabacteroides* and *Allobaculum* (Figure 4F). Principal-component analysis (PCA) showed clear differences in the microbial communities among the diet groups (Figure 4G), suggesting that a unique bacterial biomass and overall microbial community structure was established, depending on the nutritional composition of the diet.

### Dynamic effects of the gut microbiota on the plasma lipid profile

**Figure 5:**
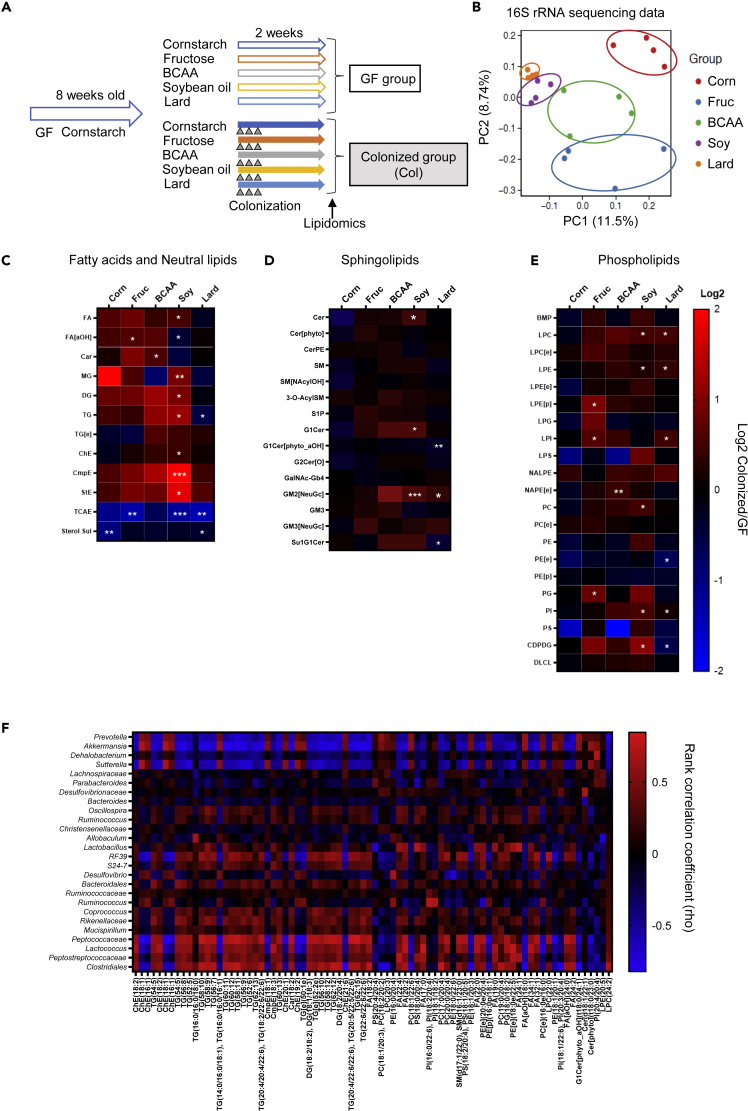
The gut microbiota established by different dietary nutrient compositions had a large impact on the plasma lipid profile (A) Schematic overview of the lipidomic study. (B) PCA analysis of the cecal microbiota compositions in the colonized (Col) mouse groups fed one of the experimental diets each for 2 weeks (10 weeks of age). (C–E) Heatmap of the class of lipid metabolites. The ratios of the levels in the Col mice to the levels in the GF mice are shown as log-2-fold changes. (C) Fatty acids and neural lipids. (D) Sphingolipids. (E) Phospholipids. The statistical significances of the differences between the GF and Col mice were determined by Student's t test (∗p < 0.05, ∗∗p < 0.01, ∗∗∗p < 0.001). (F) Heatmap of Spearman's rank correlation coefficient between individual microbial genera and plasma lipid metabolites. Excludes genera that were present at percentages of less than 1% and rho values of less than |0.7|.

To investigate the effects of the gut microbiota on the plasma lipid profile, we performed lipidomic analyses on the mice with and without intestinal bacteria. We considered it important for a precise comparison to take into account the fact that the immune system and intestinal development of GF mice are quite different from those of conventional mice. Therefore, we conducted the comparisons between 8-week-old male GF mice and their male littermates (so as to ensure that the genetic factors and physiological conditions were completely matched) colonized with the bacteria of chow-fed mice (Col mice) (Figure 5A). After 2 weeks of dietary intervention, successful colonization was confirmed by a decrease in the cecum sizes (Figure S5A) and increase in the cecal DNA levels in the colonized groups (Figure S5B). 16S rRNA sequencing of the cecal contents revealed that the colonized bacteria formed a unique structure in response to the diets with different nutritional compositions even within this period (Figures 5B, S5C, and S5D). We detected only one bacterial species of Firmicutes, *Lactococcus*, in the cecal contents of the GF mice, which was contained in the dietary casein and was killed by irradiation (Figures S5C and S5E). In the plasma lipidomic analyses, a total of 629 lipids were identified. Heatmaps showing the ratios of the lipid classes in the Col mice to those in the GF mice are shown in Figures 5C–5E. Colonization of the GF mice with the gut microbiota had little effect on the plasma lipids when the mice were fed the CS diet (Col-CS group). On the other hand, the Col-SO group, showed the largest increases in the levels of lipids, including FA, monoacylglycerol, diacylglycerol (DG), triacylglycerol (TG), cholesterol ester (ChE), campesterol ester (CmpE), and sitosterol ester (StE) in the presence of the gut microbiota. However, nearly none of these changes were observed in the Col-Lard group, and the TG levels were decreased when compared with the effects observed in the Col- SO group (Figure 5C). To understand the lipid contents in each diet, we performed lipidomic analysis of the diets (Figure S6). Overall, the FA contents were higher in the Lard diet than in the SO diet, and the DG and TG contents were high in both the diets. The discrepancies between the lipid contents in the diet and plasma lipid responses in the Col mice indicate the important interaction of the gut microbiota with the regulation of the host plasma lipids. As CmpE and StE are present in abundance in the SO diet (Figure S6), it is presumed that they are absorbed into the circulation in a gut microbiota-dependent manner. The plasma level of a primary bile acid ester, tauro-cholic acid ester (TCAE), was decreased in all the colonized diet groups when compared with the GF mice (Figure 5C). This may be because the gut microbiota promotes the conversion of primary bile acids to secondary bile acids. The SO diet also increased the plasma levels of sphingolipids, such as ceramide and the ganglioside GM2 (Figure 5D). Plasma levels of GM2 were also increased in the Col-Lard group. As GM2 is not contained in the diet (Figure S6), our findings suggest that GM2 synthesis was promoted by bacteria whose populations were specifically increased by the high-fat diets. The Fru diet also had an impact on the plasma levels of FAs and phospholipids, such as lysophosphatidylethanolamine (LPE), lysophosphatidylinositol (LPI), and phosphatidylglycerol (PG) (Figures 5C and 5E). These lipid levels in the Fru group were comparable to the levels in the CS and BCAA groups (Figure S6), suggesting that the changes in the levels of these lipids are due to the effects of fructose-derived bacteria. Next, we investigated the correlation between the lipids and bacteria and found that the presence of *Prevotella*, *Akkermansia*, *Dehalobacterium,* and *Sutterella* showed negative correlations with the plasma levels of various TGs, FAs and phosphatidylcholines (PCs), and the presence of Peptococcaceae, *Lactococcus*, Peptostreptococcaceae*,* and Clostridiales showed positive correlations with these parameters (Figure 5F). These results indicate that the gut microbiota, which are established in a nutrient-dependent manner, have differential impact on the plasma lipid profile. The SO diet-derived bacteria, which induced the highest degree of insulin resistance, had the greatest effects on various types of lipids, which may contribute to the impaired glucose metabolism and late-onset obesity observed in the SO group of mice.

As a possible mechanism, we investigated the effects of the microbiota on FA transporters in the small intestine. When compared with the GF mice, the colonized mice that were transplanted with the microbiota showed increased expression of *CD36* that facilitates FA uptake and decreased the expressions of some sterol transporters, such as *ABCA1*, *ABCG5*, and *ABCG8* (Figure S7A). Comparison of each diet group showed that Fru increased the *CD36* and *LPL* expression levels in the jejunum, whereas the SO and Lard diets decreased the expressions of these molecules in the ileum (Figures S7B and S7C), suggesting that both the microbiota and the dietary composition can contribute to regulation of the expressions of FA transporters in the small intestine.

### Gut microbiota established by the SO diet had an enormous impact on the plasma lipid profile

**Figure 6:**
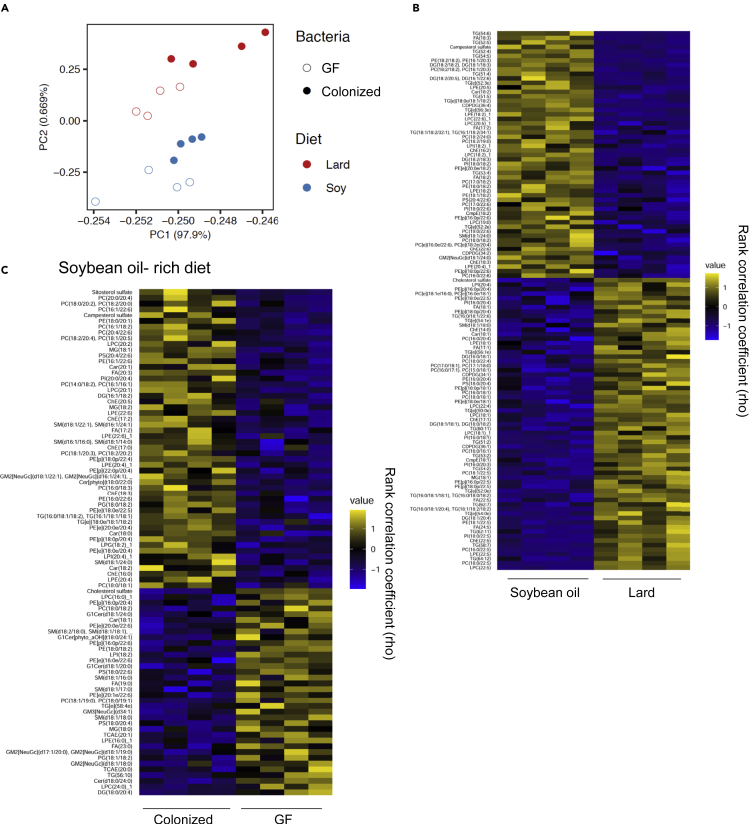
The gut microbiota established by the SO diet had a significant impact on the plasma lipid profile (A) PCA analysis of the plasma lipid metabolites in the GF and Col mouse groups fed the SO diet or Lard diet for 2 weeks. (B) Heatmap showing lipids that were significantly different between the Col mouse groups fed the SO diet or Lard diet, followed by the Benjamini-Hochberg post-test (p < 0.05, q < 0.05). (C) Heatmap showing lipids that were significantly different between the Col mice and GF mice fed the SO diet, followed by the Benjamini-Hochberg post-test (p < 0.05).

Our study revealed that the effects on the plasma lipid profile varied greatly in the presence and absence of the gut microbiota. To understand the lipid changes in greater detail, we focused on and compared the two high-fat diets. PCA of the results of lipidomic analysis showed that the lipid compositions were clearly separated among the dietary groups, and that colonization significantly changed the overall lipid profile (Figure 6A). A heatmap of the lipids that showed the greatest differences between the Col mice fed the SO and Lard diets are shown in Figure 6B. The SO diet has a high content of FAs, such as C18:2 linoleic acid and C18:3 linolenic acid, and large amounts of diacylglycerol and phospholipids containing these FAs were detected (Figures 6B and S1). Similarly, the high content of CmpE in the SO diet was also reflected in the plasma. Furthermore, because lard contains more saturated FAs, such as C16:0 palmitic acid and C18:0 stearic acid, than SO, high levels of lipid classes containing these FAs were observed in the mice fed the Lard diet (Figures 6B and S1). Therefore, in the presence of the gut microbiota, some of the lipids in the diet are likely to be reflected in the plasma.

We found that the SO diet induced a greater degree of insulin resistance than the Lard diet, and the only difference between the two diets was in the lipid composition. Furthermore, because the glucose intolerance induced by the SO diet was microbiota-dependent, we analyzed the lipids that are regulated by the gut microbiota. As shown in Figure 6C, clear changes in the levels of multiple lipids were observed in the SO diet-fed mice with or without microbiota. For example, when compared with the GF mice, the Col mice showed increased levels of sitosterol, campesterol sulfate, some PC, phosphatidylethanolamines, ChE, and TG, which contain C18:1 oleic acid, C18:2 linoleic acid, and C16:1 palmitoleic acid, suggesting the important role of the gut microbiota in regulating these lipid classes. In addition, colonization of the gut microbiota decreased the levels of some other lipids, such as cholesterol sulfate, phospholipids with saturated FAs, monohexosylceramide (G1Cer), and TCAE, suggesting that the gut microbiota established by the SO diet could have a negative impact on the appearance of these lipids in the plasma. Thus, the gut microbiota both positively and negatively regulate the lipids in the plasma. In particular, the gut microbiota established by the SO diet had the largest impact on the plasma lipids, suggesting that this could be involved in the high degree of insulin resistance observed in these mice.

## Discussion

Dietary nutrients are the major determinants of metabolism, and excessive lipid intake is known to be closely associated with the development of obesity and insulin resistance. The compositions of the gut microbiota and their activities are also controlled by the dietary nutrient compositions, besides the intestinal microenvironment. Gut microbiota have the ability to metabolize lipids with their enzymes, which affects the lipid profile of the host circulation (Staley et al., 2017). However, because the plasma lipid profile represents the overall outcome of modification of the gut microbiota, absorption of metabolites, and the metabolic balance of organs such as the liver and adipose tissue, the precise contribution of the gut microbiota still remains to be fully understood. As the intestinal microenvironments of GF mice are different from those of the conventional SPF mice, a simple comparison between these two types of mice may be affected by differences in the postnatal intestinal environments.

In this study, the experimental diets we used contained one of the three major nutrients in a relatively high proportion: carbohydrates (CS and fructose diets), proteins (BCAA diet), or lipids (SO and Lard diets). Among the major findings of the study was that both the high-fat diets (the SO and Lard diets) caused obesity and glucose intolerance with insulin resistance. However, the glucose intolerance in the SO group was worse than that in the Lard group, which was reproduced by FMT to GF mice, suggesting that the gut microbiota established by the SO diet had a more severe adverse impact on the host glucose metabolism than that established by the Lard diet. On the other hand, no significant difference was observed in the degree of chronic inflammation, the plasma leptin and ghrelin levels, or food intake between the SO and Lard groups, suggesting that there are mechanisms other than chronic inflammation and appetite regulation that might also have contributed to the worse glucose metabolism in the SO group. Plasma lipidomic analyses in GF mice and their littermates colonized with bacteria (Col mice) at the start of a 2-week dietary intervention revealed that the plasma lipid profiles were significantly affected by the presence of the gut microbiota established in response to different nutrient compositions. The SO diet increased the plasma levels of multiple types of lipids, including campesterol, sitosterol, neutral lipids, and PCs in the presence of the gut microbiota, whereas the effects of the Lard diet, also a high-fat diet, were not identical. The Col mice fed the Fru diet (Col-Fru group) showed higher plasma levels of phospholipids, such as LPE, LPI, PG, and alpha-hydroxy FA when compared with the GF mice fed the same diet (GF-Fru group), whereas those fed the BCAA diet (Col-BCAA) showed increased plasma levels of carnitine and N-acyl phosphatidylethanolamine with ether linkages when compared with the GF mice fed the same diet (GF-BCAA group). Although the percentages and major sources of lipids were similar in the CS, Fru, and BCAA diets, the three diets produced different alterations of the plasma lipid levels, presumably under the influence of the different microbiota established by the three diets, suggesting that the intestinal microbial composition and activities have unique impacts on the plasma lipid profile. The differences in the bacterial biomass and activities established by different dietary compositions may also be affected by the amount of energy intake. However, in our study, the CS and Fru or SO and Lard groups showed differences in the lipid profile derived from their microbiota despite similar levels of energy intake. Thus, not only the amount of energy intake but also differences in the nutrient compositions affect the microbial composition of the intestinal microbiota, with differential effects on the host lipid profile.

There are numerous reports about how nutrients other than lipids can also affect the plasma lipid profile and metabolism. A fructose-rich diet is generally known to cause hypertriglyceridemia (Hannou et al., 2018), insulin resistance (Huang et al., 2017), and non-alcoholic steatohepatitis (NASH) (Softic et al., 2017). Fructose also induces dysbiosis (Sellmann et al., 2015), and the addition of fructose to drinking water is known to promote hepatic lipid production via acetic acid derived from the gut microbiota (Zhao et al., 2020). The relationship between protein-rich diets and lipid metabolites is largely unknown. Patients with insulin resistance have been demonstrated to show a significant increase in BCAA production-related genes by the gut microbiota (Pedersen et al., 2016). Although BCAA is generally found in abundance in diets, it is also produced by bacteria such as *Prevotella copri* and *Bacteroides vulgatus*. Administration of *P. copri* or BCAA worsened the degree of insulin resistance in a mouse model of diet-induced obesity (Pedersen et al., 2016) (Newgard et al., 2009) (Jang et al., 2016). However, in our study, the BCAA diet group showed the greatest degree of resistance to obesity and the highest insulin sensitivity. This is probably due to the decreased food intake of the mice of the BCAA group. It has been reported that a protein-rich diet might suppress food intake via polypeptide YY (PYY) production in mouse models (Batterham et al., 2006), suggesting that the decreased food intake observed in the BCAA group in our study could be attributable to increased PYY signaling, and that we may need to consider the proportion of protein or amino acids in the diet to minimize this effect. Dietary lipids have significant effects on the gut microbiota, and the microbiota, in turn, further metabolize lipids. Rechtman et al. reported that supplementation of SO induced dysbiosis and that some bacterial taxa were positively correlated with the biomarkers of atherosclerosis. In their study, SO-based emulsion (SOE) was administered to C57B6 mice fed a chow diet for 4 weeks, and the SOE supplementation resulted in increases in the proportions of Bacteroidetes, *Mucispirillum*, *Prevotella,* and *Ruminococcus*, and decreased those of *Firmicutes*, when compared with the control group (Korach-Rechtman et al., 2020). On the other hand, Li et al. compared the changes in the microbiota of rats fed a high-fat diet containing fish oil, lard, or SO at 4% (w/w) for 3 months. In their study, they compared the lard diet with the SO diet, not the control diet, and found that the former increased the F/B ratio and the proportions of *Akkermansia* and *Tenericutes* (Li et al., 2017). In our study, the amount of SO in the SO diet was relatively high when compared with that in previously reported studies. The differential response of the microbiota to SO administration could be due to differences in the species and the experimental method.

Another research group has reported that fish oil, which is rich in polyunsaturated FAs, increased the populations of *Akkermansia*, *Lactobacilli*, and *Bifidobacteria*. On the other hand, lard, which is rich in saturated FAs, increased the populations of *Biophilia* and *Bacteroides* and caused insulin resistance by exacerbating metabolic endotoxemia (Caesar et al., 2015). In general, the ratio of intake of ω6 polyunsaturated FAs to ω3 polyunsaturated FAs is known to be correlated with the risk of development of obesity and metabolic disorders. ω6 polyunsaturated FAs, such as linoleic acid, which is contained in abundance in SO diets, has the potential to activate the arachidonic acid cascade and promote inflammation, and its bacterial metabolite, 10-hydroxy-*cis*-12-octadecenoic acid (HYA), was demonstrated to exert anti-inflammatory and anti-obesity effects in mice (Kishino et al., 2013) (Miyamoto et al., 2019). In our study, the SO diet induced chronic inflammation to the same degree or to a slightly lesser degree than the Lard diet. The inflammation may have been reduced by the anti-inflammatory effects of HYA.

The 16S rRNA sequencing analysis in our study revealed only moderate differences at the level of the phyla; however, some changes were observed at the family or genus levels and the overall community structure was unique, as shown by the PCA analysis. It is also possible that not only the bacterial proportions but also the functions and activities of the microbiota were altered. Indeed, multiple classes of lipids were significantly altered in response to the bacterial colonization; in particular, the microbiota established by the SO diet exerted the strongest influence on lipid metabolism. Plasma levels of FAs, neutral lipids, campesterol, sitosterol, all of which are contained in abundance in SO, increased significantly after the colonization. Interestingly, sphingolipids, such as ganglioside GM2, which are rarely contained in the diet, also appeared in the plasma in the presence of the gut microbiota, suggesting that the microbiota established by high-fat diets contribute to the synthesis of this lipid. This may be an important regulatory mechanism, because GM3, which belongs to the same class, is a component of the cell membrane, with important roles in cell signaling (Inamori and Inokuchi, 2020). In addition, the levels of various phospholipids were increased in the colonized group when compared with the GF group fed the SO diet (Col-SO versus GF-SO group), whereas decreases in the levels of these lipids were observed in the mice fed the Lard diet (Col-Lard group). As there were no significant differences in the phospholipid contents of the experimental diets, the microbiota have significant effects in modifying the plasma lipid levels.

It is generally known that obesity and insulin resistance induced by high-fat diets are based on chronic inflammation of the adipose tissue and liver (Lee et al., 2011) (Shoelson et al., 2006) (Saad et al., 2016). It has been shown by metagenomic analyses of the gut microbiota and gnotobiote experiments that dysbiosis of the gut microbiota is involved in this process (Cani et al., 2007). One of the factors involved is lipopolysaccharide (LPS), a component of gram-negative bacteria, which activates TLR4 and promotes inflammation. Dysbiosis induced by high-fat diets impair the intestinal barrier function and LPS flows into the circulation, resulting in so-called metabolic endotoxemia (Cani et al., 2007). In our study, both the high-fat diets (Lard and SO diets) increased the *TLR4* expressions in the liver and adipose tissue, indicating that metabolic endotoxemia was involved in the tissue inflammation and insulin resistance. However, there was no significant difference in the degree of inflammation induced between the SO group and the Lard group, suggesting that inflammation was not the sole determinant of the higher degree of insulin resistance observed in the SO diet group. Short-chain FAs, such as acetic acid, butyric acid, and propionic acid, are well-known metabolites produced by the gut microbiota such as *Prevotella* (Kovatcheva-Datchary et al., 2015), which stimulate the secretion of glucagon-like peptide-1 (GLP-1) and PYY and are known to promote insulin secretion and food intake via GPR43 and GPR41 (Tolhurst et al., 2012) (Samuel et al., 2008). In this study, we did not detect any differences in food intake between the two high-fat diet groups. In particular, transplantation of the gut microbiota from the SO diet group reproduced the impaired glucose tolerance, whereas administration of the SO diet to GF mice showed better glucose tolerance than the Lard diet. Thus, other microbial factors, such as the lipid metabolic pathways, may also contribute to the regulation of glucose metabolism.

In this study, we showed that the gut microbiota established by different nutrient compositions have unique impacts on the plasma lipid profiles of the host mice. In particular, the microbiota established by the SO diet induced severe insulin resistance, which, in turn was associated with significant effects on multiple classes of lipids. These findings have a potential to provide an evidence that long-term unbalanced diet affects human metabolic health. A more precise understanding of the interactions between the microbiota and lipid metabolism will provide new insights into how changes in the gut microbiota affect the energy and glucose metabolism.

### Resource availability

#### Lead contact

Further information and requests for resources and reagents should be directed to and will be fulfilled by the lead contact, Shiho Fujisaka (shihof@med.u-toyama.ac.jp).

#### Material availability

This study did not generate new unique reagents.

#### Date and code availability

The data that support the findings of this study are available from the lead contact upon reasonable request.

## Methods

All methods can be found in the accompanying Transparent methods supplemental file.

## Supplemental information

Document S1. Transparent methods, Figures S1–S7, and Table S1
