# Supplementary material for: Gut microbiota, determined by dietary nutrients, drive modification of the plasma lipid profile and insulin resistance
Source: iScience. 2021 Apr 16;24(5):102445. doi: 10.1016/j.isci.2021.102445 (PMC8105675; doi:10.1016/j.isci.2021.102445)
Supplement: Document S1. Transparent methods, Figures S1–S7, and Table S1 [file mmc1.pdf]

iScience, Volume *24*

Supplemental information

Gut microbiota, determined by dietary nutrients, drive modification of the plasma lipid profile and insulin resistance

Yoshiyuki Watanabe, Shiho Fujisaka, Kazutaka Ikeda, Masaki Ishikawa, Takahiro Yamada, Allah Nawaz, Tomonobu Kado, Takahide Kuwano, Ayumi Nishimura, Muhammad Bilal, Jianhui Liu, Kunimasa Yagi, Koji Hase, and Kazuyuki Tobe

Supplementary Figure 1

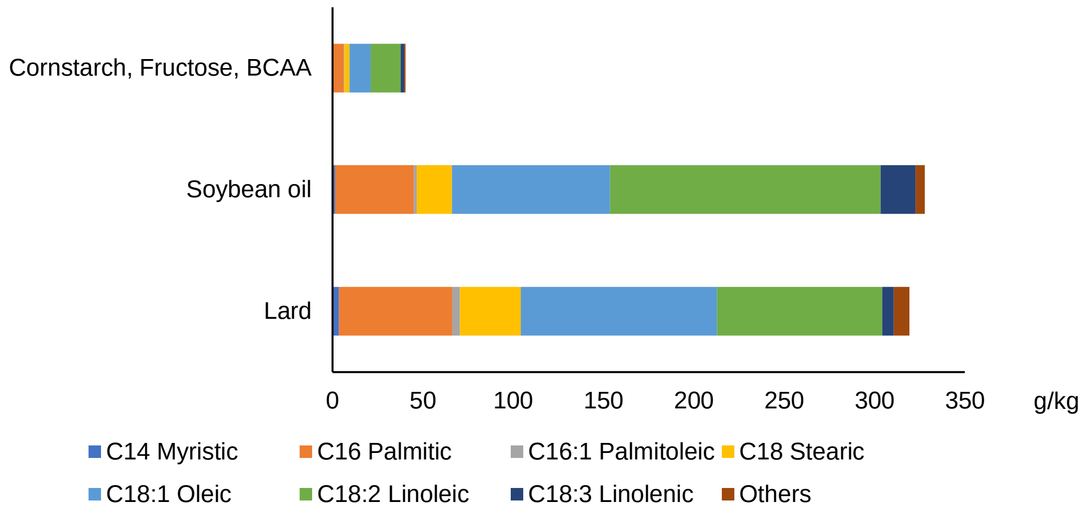

Supplementary Figure 1. Fatty acid compositions of each of the experimental diets.

Supplementary Figure 2

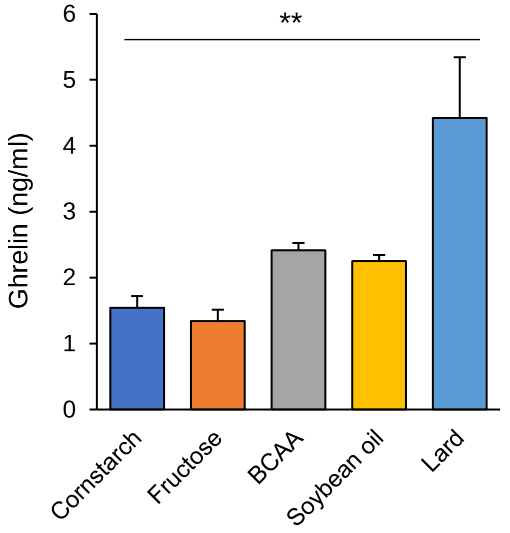

Supplementary Figure 2. Plasma ghrelin levels in the mouse groups fed one of the experimental diets each for 21 weeks. (n = 4–5/group). ***p < 0.01 by ANOVA, followed by the Tukey-Kramer test.

Supplementary Figure 3

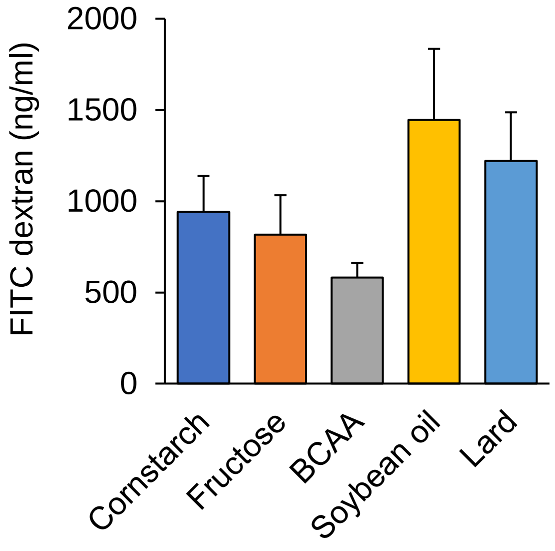

Supplementary Figure 3. Serum FITC levels in the C57B6/J mouse groups fed one of the experimental diets each, 4 hours after administration of FITC-dextran by gavage (n = 4–6).

Supplementary Figure 4

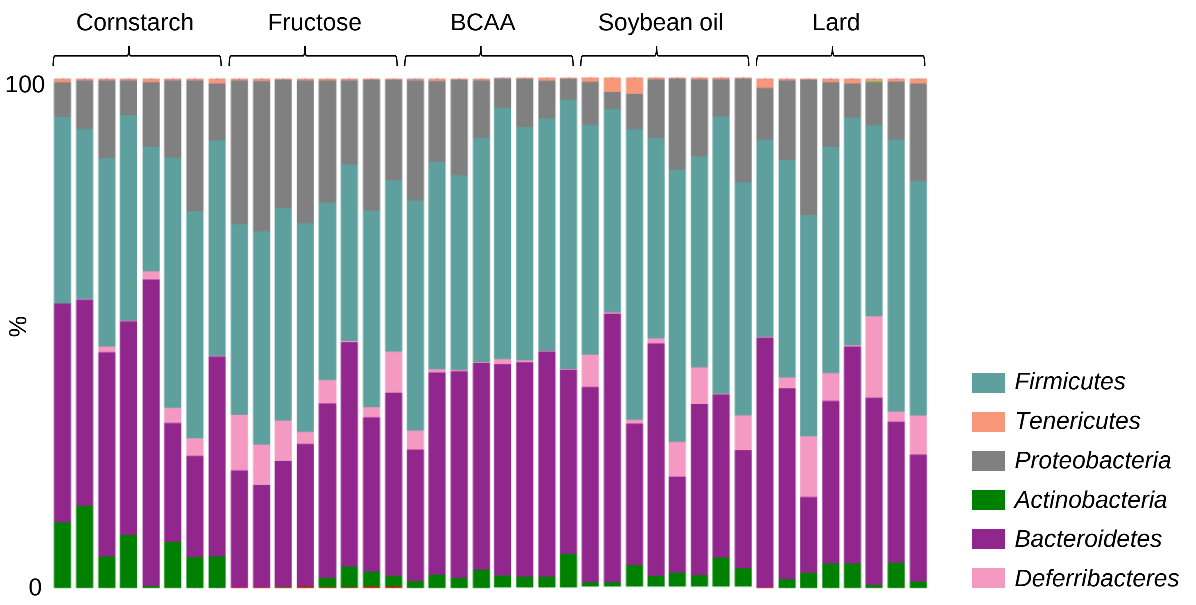

Supplementary Figure 4. Individual microbial compositions at the level of the phyla.

Supplementary Figure 5

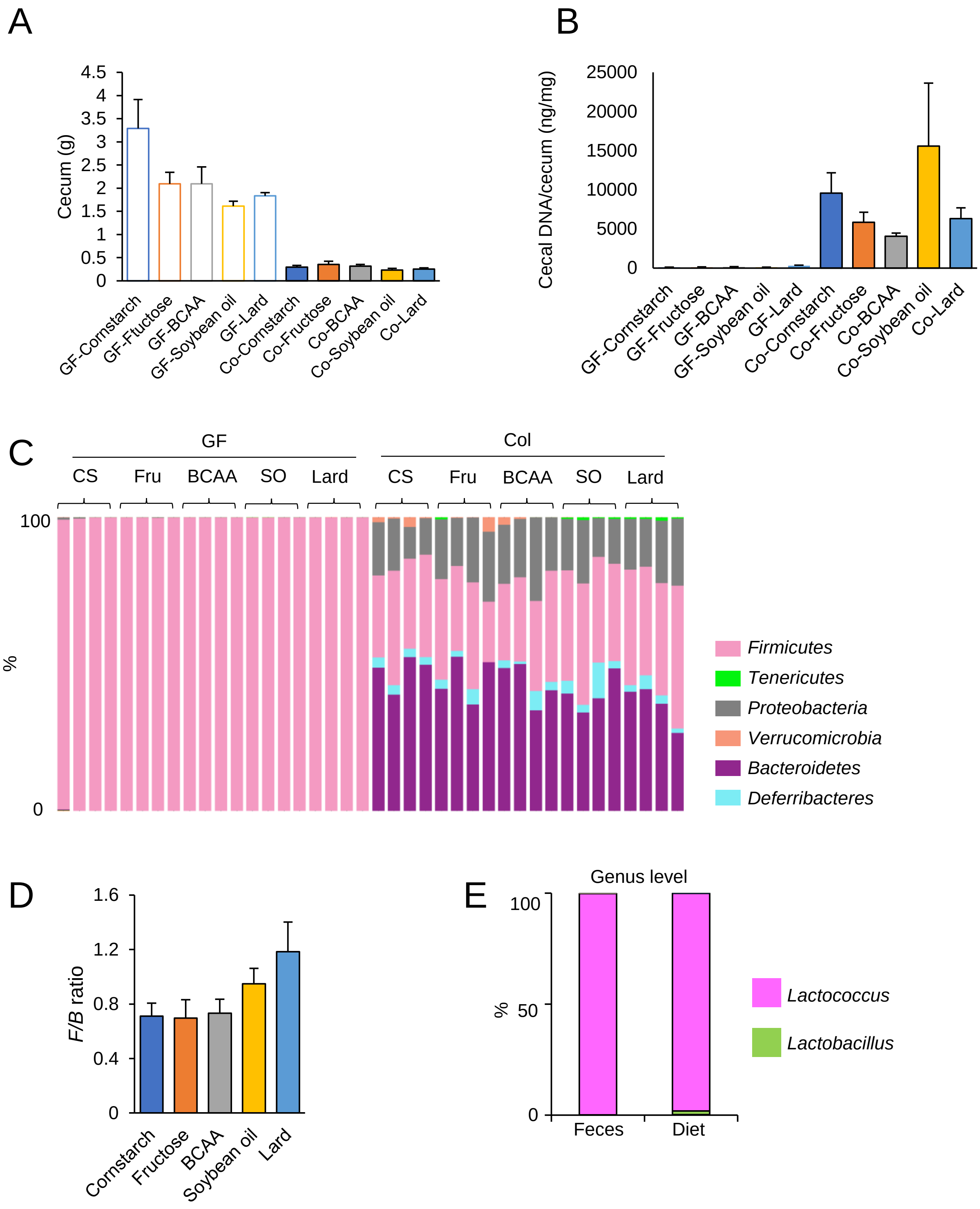

Supplementary Figure 5. (A) Cecum weights in the GF and Col mouse groups fed each of the experimental diets for 2 weeks (10 weeks of age) (n = 4). (B) Cecal DNA/cecum weights of the GF and Col mouse groups fed one of the experimental diets each for 2 weeks (10 weeks of age) (n = 4). (C) Individual microbial compositions at the level of the phyla in the cecal contents. (D) Average *Firmicutes/Bacteroides* ratio (*F/B* ratio) in the Col mice (n = 4). (E) Relative abundance of bacteria obtained by 16S rRNA sequencing at the level of the genera in the CS diet and feces of the GF mice in a vinyl isolator fed the CS diet.

Supplementary Figure 6

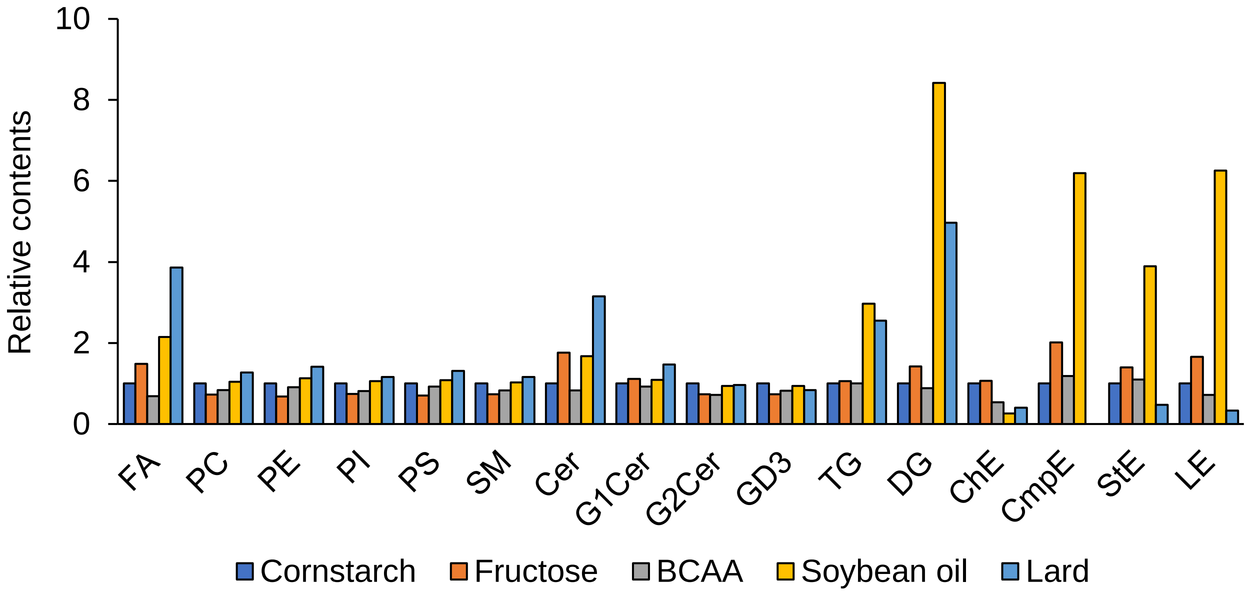

Supplementary Figure 6. Relative lipid contents in the experimental diets as assessed by lipidomic analysis.

Supplementary Figure 7

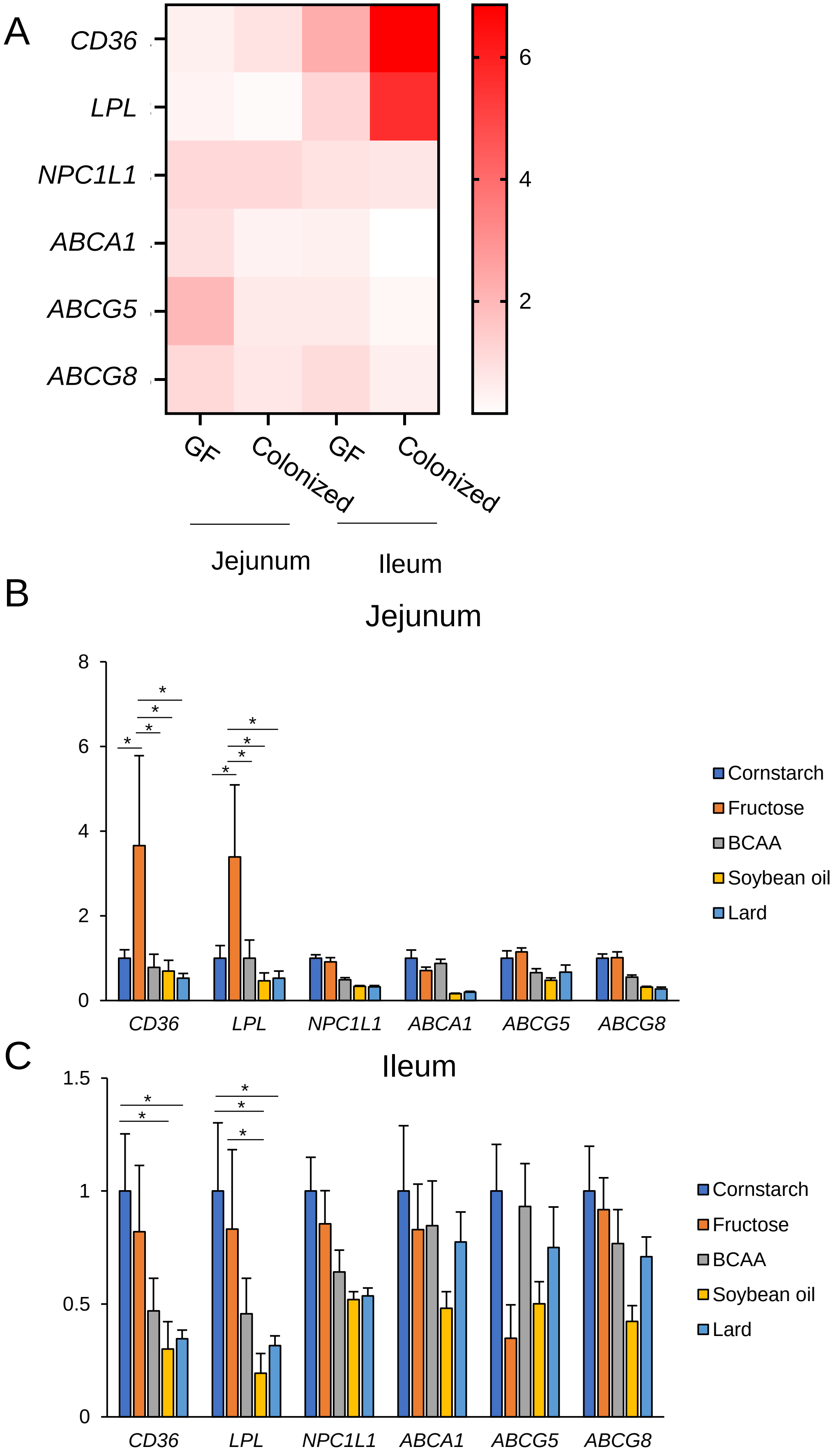

Supplementary Figure 7. (A) Heatmap of the relative expressions of lipid tranporter-related genes obtained by QPCR in the jejunum and ileum of GF and Col mice (n= 3-4). (B-C) QPCR of lipid transporter-related genes in the jejunum (B) and ileum (C) of conventional (Conv) mouse groups fed one of the experimental diets each for 2 weeks (n = 4–6).

Supplementary Table

sybrgreen	Sequence	company
β-actin	Forward:GCCGGGACCTGACAGACTAC<br>Reverse: AACCGCTCGTTGCCAATAGT	Thermo Fisher Scientific
CD11c	Forward:TGTTTGAGTGTCAGGAGCAGG<br>Reverse: GGTCACCTAGTTGGGTCTTG	Thermo Fisher Scientific
CD206	Forward:AAACACAGACTGACCCTTCCC<br>Reverse: GTTAGTGTACCGCACCCTCC	Thermo Fisher Scientific
TLR4	Forward: ACCAGGAAGCTTGAATCCCT<br>Reverse: TCCAGCCACTGAAGTTCTGA	Thermo Fisher Scientific
TNFα	Forward: ACGGCATGGATCTCAAAGAC<br>Reverse: AGATAGCAATCGGCTGACG	Thermo Fisher Scientific
Ctgf	Forward: AGCTGACCTGGAGGAAAACA<br>Reverse: CCGCAGAACTTAGCCCTGTA	Thermo Fisher Scientific
GAPDH	Forward:GTCAACGGATTTGGTCGTATTGGG<br>Reverse: TGCCATGGGTGGAATCATATTGG	Thermo Fisher Scientific
IL-1β	Forward: GCAACTGTTCCTGAACTCAACT<br>Reverse: ATCTTTTGGGGTCCGTCAACT	Thermo Fisher Scientific
IL-6	Forward: TAGTCCTTCCTACCCCAATTTCC<br>Reverse: TTGGTCCTTAGCCACTCCTTC	Thermo Fisher Scientific
iNOS	Forward: CAGCTGGGCTGTACAAACCTT<br>Reverse: CATTGGAAGTGAAGCGTTTCG	Thermo Fisher Scientific
CD11c	Forward: TGTTTGAGTGTCAGGAGCAGG<br>Reverse: AGGTCACCTAGTTGGGTCTTG	Thermo Fisher Scientific
CD206	Forward: AAACACAGACTGACCCTTCCC<br>Reverse: GTTAGTGTACCGCACCCTCC	Thermo Fisher Scientific
F4/80	Forward: CTGGGATCCTACAGCTGCTC<br>Reverse: AGGAGCCTGGTACATTGGTG	Thermo Fisher Scientific
UniF340	ACTCCTACGGGAGGCAGCAGT	Thermo Fisher Scientific
UniR514	ATTACCGCGGCTGCTGGC	Thermo Fisher Scientific
CD31	Forward: AGCTAGCAAGAAGCAGGAAGGACA<br>Reverse: TAAGGTGGCGATGACCACTCCAAT	Thermo Fisher Scientific
PDGFRα	Forward: AGCAGGCAGGGCTTCAACGG<br>Reverse: ACACAGTCTGGCGTGCGTCC	Thermo Fisher Scientific
VEGFα	Forward: AAAAACGAAAGCGCAAGAAA<br>Reverse: TTTCTCCGCTCTGAACAAGG	Thermo Fisher Scientific
IL-17	Forward: CAGGGAGAGCTTCATCTGTGT<br>Reverse: GCTGAGCTTTGAGGGATGAT	Thermo Fisher Scientific
OCC1	Forward: ACTATGCGGAAAGAGTTGACAG<br>Reverse: GTCATCCACACTCAAGGTCAG	Thermo Fisher Scientific
ZO-1	Forward: TTTTTGACAGGGGGAGTGG<br>Reverse: TGCTGCAGAGGTCAAAGTTCAAG	Thermo Fisher Scientific
Lyz-1	Forward: TCAGCTGTGTCTTCAGGGGT<br>Reverse: CCTCCAGCCAAAAGTTCAAA	Thermo Fisher Scientific
Cnr1	Forward: CTGATGTTCTGGATCGGAGTC<br>Reverse: CAGCCGTTTCTGGACAGGAGTTCTGG	Thermo Fisher Scientific
DefA	Forward: GGTGATCATCAGACCCCAGCATCAGT<br>Reverse: AAGAGACTAAAACTGAGGAGCAGC	Thermo Fisher Scientific
CD36	Forward: ACAGCTGCCTTCTGAAATGTGTGGA<br>Reverse: TTTTCTACGTGGCCCGGTTCTAATTCA	Thermo Fisher Scientific
LPL	Forward: TCAGCTGTGTCTTCAGGGGT<br>Reverse: TTTGGCTCCAGAGTTTGACC	Thermo Fisher Scientific
NCP1L1	Forward: CCTAAAGGGGGCCTAGCAGC<br>Reverse: TGCCCGGAGAGCTTCTGTAA	Thermo Fisher Scientific
ABCA1	Forward: GGAAGGGACAAATTGTGCTG<br>Reverse: TGCACAAGGTCCTGAGAATG	Thermo Fisher Scientific
ABCG5	Forward: AGCCATTCACAAACACCTCC<br>Reverse: TCCTTGTACATCGAGAGTGGC	Thermo Fisher Scientific
ABCG8	Forward: TACTTCAGGATGCTTCGCAGG<br>Reverse: TGCTATGAGACCTCCAGGGT	Thermo Fisher Scientific

Supplementary Table. List of target genes for quantitative RT-PCR. Related to Figure 2, Figure 3, Figure 4 and Supplementary Figure 7.

EXPERIMENTAL MODEL AND SUBJECT DETAILS

Mouse procedures

Male C57BL/6 mice were purchased from Japan SLC, Inc. (Japan). Germ-free (GF) C57BL/6 mice were housed in vinyl isolators and obtained by natural mating for this study. The mice were maintained under a 12-hour light-dark cycle and were given free access to water and food. Eight-week-old mice were divided into 5 dietary groups that were fed a diet rich in cornstarch (CS), fructose (Fru), branched chain amino acids (BCAA), soybean oil (SO), or lard (Lard) (Research Diets Inc. NJ, USA). The nutrient composition of each of the diets is shown in Table and Supplementary Figure 1. Unless stated otherwise, male mice were used. The mice were fed *ad libitum*. Each diet was stored frozen until use and replaced weekly with fresh diet. The amount of energy intake was calculated by measuring the average weight of food consumed and multiplying it by the number of calories per unit weight of diet. To fully examine the reproducibility of the experiment, we conducted experiments in several independent cohorts and saw the same trends with reproducibility. For studies involving measurement of relatively large variations, such as the body weight, food intake, and glucose tolerance test results, data from several cohorts of mice are displayed by verification considering the cage effect. In some experiments, the insulin tolerance test and sacrifice of mice were performed at different weeks of age, we have displayed the data from one representative cohort without mixing the results with those from other cohorts. In the study using GF mice, the number of mice per group varied, because mice bred in our isolator were used in order to ensure that the environmental and genetic backgrounds and ages of all the mice were completely matched. The animal care policies and procedures for the experiments were approved by the Animal Experiment Committee of the University of Toyama.

METHOD DETAILS

Oral glucose tolerance test (OGTT) and insulin tolerance test (ITT)

Each of the OGTT (2 g/kg weight) and intraperitoneal ITT (1.0 unit/kg) was performed after the animals had been denied access to food for a 4-hour period. Blood samples were collected from

the tail at specific time-intervals and the glucose levels were measured using Stat Strip XP3 (Nipro, Japan).

Analysis of the plasma levels of insulin, leptin, ghrelin and the liver TG content

Plasma levels of insulin, leptin and ghrelin were measured using the Insulin ELISA Kit (Shibayagi, Japan), Leptin ELISA Kit (Morinaga, Japan), and Ghrelin Enzyme Immunoassay Kit (RayBiotech, U.S.A.), respectively, according to the manufacturer’s instructions. The plasma TG levels and liver TG contents were measured with the Triglyceride Colorimetric Assay Kit (Cayman Chemical Company, U.S.A.), according to the manufacturer’s instructions.

RNA isolation and real-time polymerase chain reaction (RT-PCR)

Tissues for RT-PCR were collected and preserved in RNAlater solution from Ambion (Austin, Texas), according to the manufacturer’s instructions. Total RNA was extracted with the RNeasy kit, (Qiagen, Hilden, Germany) and reverse-transcribed using the TaKaRa PrimeScript RNA Kit, cat# RR036A (Takara, Japan), according to the manufacturer’s instructions. Quantitative PCR was performed using the TaqMan method (1 cycle at 50°C for 2 min, 95°C for 10 min, and 40 cycles at 95°C for 15 s, 60°C for 1 min) or the SYBR Green method (1 cycle at 95°C for 30 s, and 45 cycles at 95°C for 10 s and 60°C for 20 s). Each sample was run in duplicate and the relative mRNA levels were calculated using the standard curve method and normalized to the mRNA levels of *β-actin* or *GAPDH*. The primer sequences used are listed in Supplementary Table.

Flow-cytometric analysis

Isolation and separation of the stromal vascular fractions (SVF) of the eWAT and liver, and subsequent flow-cytometric analysis was performed as previously described (Fujisaka et al., 2009) (Fujisaka et al., 2016). Live cells in the SVF were collected for further analysis after exclusion of dead cells by gating with 7AAD. M1 and M2 macrophages were identified as CD45-positive/F4/80-positive/CD11c-positive/CD206-negative and CD45-positive/F4/80-positive/CD11c-negative/CD206-positive cells, respectively. Macrophages in the liver were identified as CD45-positive/F4/80-positive cells. Flow-cytometric analysis was performed with the FACSDiva Version 6.1.2 automated cell analyzer, FACSCanto II (BD Biosciences, San Jose, CA). The data analyses were performed using the FlowJo software (Tree Star, Ashland, OR). Unstained specimens and a fluorescence minus one (FMO) control were used to justify the gating strategy. Antibody information is shown in KEY RESOURCE TABLE.

FITC-dextran experiment

The FITC-dextran experiments were performed as described previously (Fujisaka et al., 2016). FITC-dextran (Sigma-Aldrich. U.S.A.) was administered orally. Four hours after the gavage, blood samples were collected by cardiac puncture. The plasma was diluted 1:1 (vol/vol) in PBS and the fluorescence intensity of each sample was measured (excitation: 485 nm, emission: 528 nm) with a fluorescence spectrometer. The FITC-dextran concentrations were calculated from a standard curve.

16S rRNA sequencing analysis

DNA was extracted from the mouse cecal contents or feces using a QIAmp Powerfecal DNA kit (QIAGEN, CA). A multiplexed amplicon library converting the 16S rDNA gene V4 region was generated from the DNA samples and the sequencing was performed in Bioengineering Lab. Co., Ltd. (Kanagawa, Japan). Principal component analysis (PCA) was performed using the prcomp command of R version 3.2.1. The database was Greengene's 97 OTU attached to the microbiota analysis pipeline, Qiime.

Fecal microbiota transplantation (FMT)

For the bacterial transfer experiment into GF mice, FMT was performed 3 times every other day by gastric gavage of 200 μl of filtered feces suspended in saline. The fecal samples were collected from groups of mice that had been fed the CS, Fru, BCAA, SO or Lard diet for 18 weeks. All the recipient mice were then kept on the CS diet after the transfer.

For the bacterial transfer experiment into conventional SPF mice, 9-week-old mice were treated with a mixture of vancomycin (0.5g/L), metronidazole (1g/L), neomycin (1g/L) and ampicillin (1g/L) (Sigma-Aldrich, St. Louis, MO) in drinking water for 3 days prior to the transfer. Fecal microbiota transfer was performed by gastric gavage of 200 μl of filtered feces suspended in saline. The fecal samples were collected from groups of mice that had been fed the SO or Lard diet for 18 weeks. The recipient mice were then kept on the Lard diet after the transfer.

For colonization of GF mice for lipidomic study, fresh fecal samples, suspended in saline, were collected from purchased 5-week-old male C57BL/6J mice in a container that had been fed a standard diet. Then the fecal microbiota transfer was performed into 8-week-old GF mice 3 times every other day. The recipient mice were kept on the CS, Fru, BCAA, SO, or the Lard diet after

the transfer.

Lipidomic analysis

Lipid extraction and non-targeted lipidomics were performed according to previously described methods (Ikeda, 2015) (Tsugawa et al., 2017), with a few modifications. In brief, total lipids from 12 μl of plasma were extracted using EquiSPLASH (Avanti Polar Lipids, Inc., Alabaster, AL, U.S.A.) and palmitic acid-1,2-13C2-palmitic acid (Merck, Darmstadt, Germany) as internal standards. Samples were separated on an L-column3 C18 column (50 × 2.0 mm i.d., particle size 2.0 μm, CERI; Saitama, Japan) at a flow rate of 300 μl min^{-1} at 45°C, using the ACQUITY UPLC system (Waters, Milford, MA, U.S.A.) equipped with a binary pump and automatic sample injector. Solvent A consisted of acetonitrile/methanol/water (1:1:3, v/v/v) and solvent B consisted of isopropanol, both containing 5 mM of ammonium acetate. The LC gradient conditions were as follows: 0–6.5 min, 0%–64.0% B; 6.5–13.5 min, 64.0%–76.5% B; 13.5–18.0 min, 76.5%–98% B; 18.0–20.0 min, held at 98%. Then, the column was re-equilibrated with 100% A for 5 min. Qualitative and quantitative analyses of lipids were performed by MS and data-dependent MS/MS acquisition over a scan range of m/z 140–1700, using the Triple TOF 6600 System (AB SCIEX, Framingham, MA, U.S.A.) in the negative and positive ion modes. Raw data files from the TOF-MS were converted to MGF files using the program AB SCIEX MS converter, for subsequent quantitative analysis with 2DICAL (Mitsui Knowledge Industry, Tokyo, Japan). Identification of the molecular species was accomplished by comparison with the retention times and MS/MS spectra of commercially available standards or reference samples.

The heat maps were generated with the GraphPad Prism8 software (GraphPad Software, San Diego, CA, U.S.A.) using the mean fold changes in the sum of the ion peak heights of all the lipid molecules within each class calculated as the ratios in the Col to the GF mice. Statistical significances of the differences between the GF and Col mice were determined by Student's t-test (*p < 0.05, **p < 0.01, ***p < 0.001).

Abbreviations

FA, fatty acid; FA[aOH], alpha-hydroxy fatty acid; Car, acylcarnitine; MG, monoacylglycerol; DG, diacylglycerol; TG, triacyl glycerol; TG[e], ether-linked TG; ChE, cholesteryl ester; CmpE, campesteryl ester; StE, stigmasteryl ester; TCAE, taurocholic acid ester; Sterol Sul, sterol sulfates; BMP, bismonophosphotidate; LPC, lysophosphatidylcholine; LPC[e], ether-linked LPC; LPE, lysophosphatidylethanolamine; LPE[e], ether-linked LPE; LPE[p], vinyl ether-linked LPE; LPG, lysophosphatidylglycerol; LPI, lysophosphatidylinositol; LPS, lysophosphatidylserine; NALPE, N-acyl lysophosphatidylethanolamine; NAPE[e], N-acyl phosphatidylethanolamine with ether linkages; PC, phosphatidylcholine; PC[e], ether-linked PC; PE, phosphatidylethanolamine; PE[e], ether-linked PE; PE[p], vinyl ether-linked PE; PG, phosphatidylglycerol; PI, phosphatidylinositol; PS, phosphatidylserine; CDPDG, cytidine diphosphate-diacylglycerol; DLCL, dilysocardiolipin; Cer, ceramide; Cer[phyto], phytoceramide; CerPE, ceramide phosphatidylethanolamine; SM, sphingomyelin; SM[NAcylOH], SM with N-hydroxy fatty acyl residues ; 3-O-AcylSM, SM with N-3-acylated fatty acyl residues ; S1P, sphingosine-1-phosphate; G1Cer, monohexosylceramide; G1Cer[phyto_aOH], alpha-hydroxy monohexosylphytoceramide; G2Cer[O], oxidized dihexosylceramide; Gb5, globopentaose; GM2[NeuGc], ganglioside GM2 containing N-glycolyl neuraminic acid; GM3, ganglioside GM3; GM3[NeuGc], ganglioside GM3 containing N-glycolyl neuraminic acid; Su1G1Cer; cerebroside sulfate; Taurine, acyl-taurine

QUANTIFICATION AND STATISTICAL ANALYSIS

All the values presented are the means ± SEM. Statistical significances of differences among groups were determined by the two-tailed unpaired Student's t-test (two groups) or ANOVA followed by the Tukey-Kramer post hoc test or Dunnett post hoc test (three or more groups). The tests were adjusted for multiple testing using the Benjamini-Hochberg false discovery rate, where appropriate. $P < 0.05$ was considered as being indicative of statistical significance.

KEY RESOURCES TABLE

REAGENT or RESOURCE	SOURCE	IDENTIFIER
Antibodies		
CD45 Antibody	Thermo Fisher Scientific	25-0451-82
APC/Cyanine7 anti-mouse F4/80	Thermo Fisher Scientific	#123118
PE Hamster Anti-Mouse CD11c	Thermo Fisher Scientific	#553802
Rat anti Mouse CD206:Alexa Fluor® 647	BIO RAD	MCA2235A647
7-AAD	Thermo Fisher Scientific	#51-68981E
Chemicals, peptides, and recombinant proteins		
Ampicillin	Sigma-Aldrich	#019M4771V
Neomycin	Sigma-Aldrich	#SLBN5615V
Metronidazole	Sigma-Aldrich	#MKCJ4156
Vancomycin	LKT LAB	#V0252
Critical commercial assays		
Rneasy Mini Kit (50)	QIAGEN	74104

5x Primescript RT Master MIX perfect Realtime	Takara Bio	RR036A
PowerFecal DNA kit	QIAGEN	12830-50
Triglyceride Colorimetric Assay	Cayman	10010303
Insulin ELISA Kit	Shibayagi	MIT-769
Mouse Ghrelin Enzyme Immunoassay Kit	RayBio	EIAM-GHR
Morinaga Mouse/Rat Leptin ELISA Kit	MIoBS	M1305
TB Green Fast qPCR Mix	Takara Bio	RR430A
Fluorescein isothiocyanate–dextran	Sigma-Aldrich	46944
Experimental models: organisms/strains		
Mouse C57BL/6	Japan SLC, Inc.	N/A
Oligonucleotides		
Primer sequences used for RT-qPCR	See Supplementaey Table	N/A
Software and algorithms		
R 3.6.2	R Development Core Team	N/A
GraphPad Prism 9	GraphPad Prism	N/A
JMP15	SAS	N/A
FlowJo_v10.6.1	Becton, Dickinson and Company	N/A

SUPPLEMENTAL REFERENCES

Fujisaka, S., Ussar, S., Clish, C., Devkota, S., Dreyfuss, J.M., Sakaguchi, M., Soto, M., Konishi, M., Softic, S., Altindis, E., et al. 2016. Antibiotic effects on gut microbiota and metabolism are host dependent. J Clin Invest 126, 4430-4443.

Fujisaka, S., Usui, I., Bukhari, A., Ikutani, M., Oya, T., Kanatani, Y., Tsuneyama, K., Nagai, Y., Takatsu, K., Urakaze, M., et al. 2009. Regulatory mechanisms for adipose tissue M1 and M2 macrophages in diet-induced obese mice. Diabetes 58, 2574-2582.

Ikeda, K., 2015. Mass spectrometric analysis of phospholipids by target discovery approach. Bioactive Lipid Mediators 25, 349–356.

Tsugawa, H., Ikeda, K., Tanaka, W., Senoo, Y., Arita, M., Arita, M., 2017. Comprehensive identification of sphingolipid species by in silico retention time and tandem mass spectral library. J. Cheminform. 9, 19.
